# Roles of KChIP1 in the regulation of GABA-mediated transmission and behavioral anxiety

**DOI:** 10.1186/1756-6606-3-23

**Published:** 2010-08-02

**Authors:** Kun Xia, Hui Xiong, Yeonsook Shin, Danling Wang, Tom Deerinck, Hiroto Takahashi, Mark H Ellisman, Stuart A Lipton, Gang Tong, Giannina Descalzi, Dongxian Zhang, Min Zhuo, Zhuohua Zhang

**Affiliations:** 1 State Key Laboratory of Medical Genetics, Central South University, Changsha, Hunan 410078, China; 2 Burnham Institute for Medical Research, La Jolla, CA 92037, USA; 3 Department of Neurosciences, University of California, San Diego, La Jolla, CA 92093, USA; 4 National Center for Microscopy and Imaging Research, University of California, San Diego, La Jolla, CA 92093, USA; 5 Department of Physiology, University of Toronto, Toronto, ON., M5 S 1A8 Canada; 6 Department of Brain and Cognitive Sciences, Seoul National University, Seoul 151-746, Korea

## Abstract

K^+ ^channel interacting protein 1 (KChIP1) is a neuronal calcium sensor (NCS) protein that interacts with multiple intracellular molecules. Its physiological function, however, remains largely unknown. We report that KChIP1 is predominantly expressed at GABAergic synapses of a subset of parvalbumin-positive neurons in the brain. Forced expression of KChIP1 in cultured hippocampal neurons increased the frequency of miniature inhibitory postsynaptic currents (mIPSCs), reduced paired pulse facilitation of autaptic IPSCs, and decreases potassium current density. Furthermore, genetic ablation of KChIP1 potentiated potassium current density in neurons and caused a robust enhancement of anxiety-like behavior in mice. Our study suggests that KChIP1 is a synaptic protein that regulates behavioral anxiety by modulating inhibitory synaptic transmission, and drugs that act on KChIP1 may help to treat patients with mood disorders including anxiety.

## Background

Neuronal calcium sensor (NCS) proteins contain EF-hand calcium binding domains and are conserved throughout evolution [1-3]. In humans, they are encoded by 14 genes, some of which alternatively splice during transcription. Based on the order of their evolutionary appearance, NCS proteins are classified into class A to E5 subfamilies [1,4]. Four KChIP proteins constitute the fifth subfamily (Class E) and are unique to mammals. KChIPs interact with voltage-gated potassium channels and presenilins [5-8]. KChIP1, KChIP3, KChIP4 are expressed predominantly in brain, while KChIP2 is highly expressed in both heart and brain [7,9].

Frequenin, the *Drosophila *NCS-1, increases neurotransmitter release at the neuromuscular junction and has been implicated in synaptic efficacy [10]. Gene disruption of NCS-1 in *C. elegans *causes defects in associative learning and memory, suggesting an involvement in regulating synaptic plasticity [11]. Moreover, mammalian NCS-1 was recently reported to facilitate P/Q-type calcium currents at presynaptic terminals of the calyx of Held synapse [12]. Similarly, KChIPs have been suggested to function as the ß (modulatory) subunit of fast transient (A-type) potassium channels. Potassium channels are responsible in part for repolarizing the plasma membrane during action potentials [13]. Kv4 potassium channels are voltage-gated fast transient (A-type) channels that modulate firing rates and shape first spike latency. Kv1 and Kv3 subunits are found at presynaptic nerve terminals [13], whereas Kv4.2 is primarily in postsynaptic membranes [14,15]. Inactivation of KChIP3 in mouse neurons results in enhanced long-term potentiation (LTP) via down-regulation of Kv4-channel activity [16], further supporting its role in modulating potassium channels *in vivo *[8]. KChIPs, NCS-1, and frequenin interact with potassium channels (e.g., Kv4.2 and Kv4.3) modulating their trafficking and kinetic properties, suggesting that NCS proteins affect the physiological actions of potassium channels in neurons [7,8,17-27].

The present study investigated KChIP1 expression in the mouse brain and its function. KChIP1 is predominantly localized in a subpopulation of parvalbumin-positive GABAergic neurons. Patch-clamp recordings revealed that KChIP1 facilitated GABA-mediated IPSCs by increasing presynaptic transmitter release. KChIP1 over-expression decreased potassium current density whereas ablation of KChIP1 expression resulted in increased potassium current density in mouse Purkinje neurons. Furthermore, KChIP1 knockout (KO) mice exhibited enhanced anxiety-like behavior compared to wildtype (WT) mice. Our results provide the first evidence that KChIP1 plays an important role in modulating inhibitory synaptic transmission and contributes to behavioral anxiety.

## Methods

### Neuronal cell culture and reagents

Primary hippocampal and cortical neurons were dissociated from newborn or E18 rats, respectively, and maintained in culture for 1-3 weeks as described previously [28]. Primary cerebellum cultures with enriched Purkinje neurons were made as previously described [29]. Anti-KChIP1 monoclonal antibody was commercially generated [9]. Anti-SV2 was a gift from Dr. K.M. Buckley. Anti-synaptophysin, anti-calbindin and anti-enhanced green fluorescence protein (EGFP) were purchased from Sigma (St. Louis, MO) and Clontech (Palo Alto, CA), respectively.

### Plasmids

The cDNA encoding KChIP1 was cloned into pEGFPN (Clontech) and pcDNA3.1(-)MycHis (Invitrogen, San Diego, CA) by PCR to generate plasmids expressing C-terminally tagged KChIP1-EGFP and KChIP1-mycHis in mammalian cells. Primers for PCR were: 5'-gggaattcgccaccatgggggccgtcatgggcacc-3' (forward) and 5'-ggggatccacatgacattttgaaacagctggag-3' (reverse). The coding sequence of KChIP1 was cloned into pGEX4T2 (Amersham, Uppsala, Sweden) to express a GST-KChIP1 fusion protein. All plasmids were confirmed by sequencing. For expressing KChIP1 in neurons, EGFP and KChIP1-EGFP cDNAs were excised from pEGFPN2 and pEGFPN2-KChIP1 plasmids by Eco RI and Not I digestion, respectively. The fragments were then cloned into a Sma I digested pSFV1 plasmid (Invitrogen).

### Fusion protein and antibody preparation

GST-KChIP1 fusion protein was produced and purified following the manufacturer's protocol. The GST-KChIP1 protein was used to immunize rabbits in order to raise polyclonal anti-KChIP1 antibodies. Antibody production was performed by Immungenex (San Diego, CA).

### In situ hybridization

Non-radioactive *in situ *hybridization was performed essentially as described previously [14]. Briefly, adult mice (2-4 months) were perfused and fixed with 4% paraformaldehyde. Brains were dissected and fixed overnight. Fifty-micron thick cryo-cut sections were obtained. Antisense and sense RNA probes were *in vitro *transcribed and labeled using KChIP1 cDNA fragments as templates and a mixture of nucleotides containing digoxygenin-UTP. Hybridization was carried out at 65°C overnight. Signals were detected with an alkaline phosphatase-conjugated goat anti-digoxygenin antibody and developed using NBT/BCIP. Immunostaining was performed after *in situ *hybridization. Sections were analyzed under either deconvolution or confocal microscopy.

### Viral infection

We constructed a fusion protein between EGFP and KChIP1, and inserted the fused gene or EGFP alone under the CMV promoter into a Semliki Forest Virus vector (pSFV, Invitrogen). pSFV1/EGFP, pSFV1/KChIP1-EGFP, and virus Helper 2 DNA were linearized by Spe I. One μg DNA was transcribed *in vitro *using the mMessage mMachine kit (Ambion, Austin, TX). pSFV1 viral particles were generated according to instructions provided by Invitrogen. For infection of primary neurons, the original culture medium was changed to a serum-free medium. Viral particles pre-treated with chymotrypsin (Sigma) were added to the cultures at a 1 to 10 dilution. The infection was performed for 1 hour at 37°C, followed by replacement of the infection medium with conditioned culture medium. Infected neurons were monitored for expression of EGFP and used for patch-clamp recordings 18-30 h later.

### Immunoassays

Immunoprecipitation, immunoblotting, and immunofluorescence were performed essentially as described previously [30,31]. Briefly, cells were rinsed once with PBS and lysed on ice in 0.7% NP-40 buffer (10 mM HEPES, pH7.5, 142.4 mM KCl, 5 mM MgCl_2_, 1 mM EGTA, and 0.7% NP-40). Insoluble cell debris was cleared by centrifugation at 14,000 rpm at 4°C for 30 min in an Eppendorf centrifuge, and the supernatants were collected for immunoprecipitation. Antibodies for immunoprecipitation (3 μg) and protein G beads (25 μl) were added to the lysates and mixed at 4°C overnight with a nutator. Following precipitation, protein-antibody-bead complexes were washed at least three times in 0.7% NP-40 buffer. The proteins were then separated on a 4-20% Tris-Glycine gel (Invitrogen), electro-transferred to PVDF membranes (Millipore, Billerica, MA), immunodectected with appropriate antibodies, and developed using an ECL kit (Amersham). Because the molecular weight of KChIP is similar to that of the IgG light chain, immunoprecipitation of KChIP1 was performed using beads to which the antibody was immobilized by a Seiz × Mammalian Immunoprecipitation kit (Pierce, Rockford, IL). For immunofluorescence staining, cells were grown on circular cover slips in 24-well culture dishes, fixed with 3.7% paraformaldehyde for 15 min, permeabilized with 0.1% Tween-20 for 10 min, and stained with primary antibody for 1 h. This was followed by incubation with a Cy2- or Cy3-labeled secondary antibody for 1 h. Cells were washed, cover slipped in anti-fade medium (Fisher, Pittsburgh, PA), and analyzed under confocal microscopy.

Brain sections were analyzed by immunohistochemistry as follows. Adult Sprague Dawley rats were anesthetized with nembutal and perfused with PBS followed by 4% formaldehyde in PBS (pH 7.4) using intracardiac catheterization. The brain was removed and fixed for an additional hour, after which it was rinsed in PBS, and 80 μm thick vibratome sections were cut. Brain sections were permeabilized in 0.1% Triton X-100, 1% normal donkey serum, and 1% cold water fish gelatin (Sigma) in PBS for 30 min. Sections were then incubated in primary antibody for 18 h at 4°C and washed in buffer, followed by further incubation with the appropriate secondary antibody (Jackson ImmunoResearch, West Grove, PA) in PBS for 1 hr at 4°C. Sections were rinsed in PBS, mounted in Gelvatol, and examined under confocal microscopy.

### Expression and recording of Kv4.3 channels in Xenopus oocytes

cDNAs encoding Kv4.3, KChIP1, or KChIP1-EGFP were subcloned into the pCS2 plasmid. Capped RNAs were made using Message Machine RNA polymerase kits (Ambion) and verified by gel electrophoresis. Xenopus oocytes were co-injected with Kv4.3 (5 ng) and either EGFP (10 ng), KChIP1 (10 ng), or KChIP1-EGFP RNAs (20 ng), and maintained in ND96 solution (96 mM NaCl, 2 mM KCl, 1.8 mM CaCl_2_, 1 mM MgCl_2_, and 5 mM HEPES, pH 7.4). Kv4.3 potassium currents were recorded under two-electrode voltage clamp at a holding potential of -100 mV (Amplifier Model OC-725A, Warner Instrument, Hamden, CT). Recording electrodes were filled with 2 M KCl and had resistances between 0.3 and 1.0 mΩ. Currents were sampled at 5-10 kHz and filtered at 1-2 kHz. All recordings were performed at room temperature, and oocytes were perfused continuously with an external solution containing 96 mM NaCl, 2 mM KCl, 2 mM CaCl_2_, 1 mM MgCl_2_, 10 mM HEPES (pH 7.4). Data collection and analysis were performed using the pClamp 9.0 software program (Axon Instruments, Foster City, CA).

### Whole-cell patch-clamp recording

Autaptic recordings were made from somas of isolated, single hippocampal neurons in low-density culture conditions with patch pipettes (4-6 MΩ resistance) that were filled with an internal solution consisting of (in mM): 140 potassium gluconate, 17.5 KCl, 9 NaCl, 1 MgCl_2_, 10 HEPES, and 0.2 EGTA, at pH 7.4 [28,32]. The standard external solution contained 150 mM NaCl, 3 mM KCl, 10 mM HEPES, 5 mM glucose, 2 mM CaCl_2_, 50 μM D-2-amino-5-phosphonovalerate (APV), and 10 μM CNQX at pH 7.4. For the paired pulse facilitation experiments, 2 mM CaCl_2 _was replaced by 1 mM CaCl_2 _and 3 mM MgCl_2_. The currents were low-pass filtered at 2-5 kHz and digitally sampled at 10-20 kHz. Capacitative currents were subtracted and blanked. Illustrated traces represent an average of 4-8 responses. For experiments studying mIPSCs, TTX (1 μM) was added to block Na^+ ^channels and resulting action currents. For recording K^+ ^currents, we used an external solution comprised of 115 mM NaCl, 2.5 mM KCl, 1.5 mM MgCl_2_, 10 mM HEPES, and 0.1 mM BAPTA; 1 μM TTX and 3 mM CoCl_2 _were added to inhibit Na^+ ^and Ca^2+ ^currents, respectively. Potassium currents were evoked with a series of incremental voltage steps of 100 ms duration to 45 mV from a holding potential of -75 mV. Steady-state current amplitudes were measured 75 ms after the initiation of each voltage step and normalized to cell capacitance. For recordings of Ca^2+^-channel mediated Ba^2+ ^currents, the external solution contained 160 mM TEA-Cl, 2 mM BaCl_2_, and 10 mM HEPES-CsOH. In addition, 1 μM TTX was added to block Na^+ ^currents. A cesium gluconate-based solution was used as the internal solution [28]. Voltage-activated Ba^2+ ^currents were evoked by applying a 105 msec step to a test potential of 0 mV every 10 sec from a holding potential of -80 mV. The peak Ba^2+ ^currents were calculated by subtraction of Cd^2+^- sensitive Ba^2+ ^current and normalized to cell capacitance. Solution changes were made with fast-flow, gravity-fed flow tubes gated by valves. Data acquisition and analysis were made with pClamp 8 (Axon Instruments, Union City, CA) or a mini analysis program (Synaptosoft, Decatur, GA). Results are expressed as mean ± SE. All experiments were performed at room temperature.

### Behavioral measurements

Hot-plate and tail-flick test: KChIP1^-/- ^mice were generated, genotyped and breed as described[33]. In the hot-plate test, mice were placed on a standard thermal hotplate with a heated surface (55°C) (Columbus Instruments, Columbus, OH). The latency for nociceptive responses was recorded with a cutoff time of 30 seconds. The spinal nociceptive tail-flick reflex was evoked by radiant heat (Columbus Instruments, Columbus, OH) applied to the underside of the tail, and latencies were measured with a cutoff time of 10 seconds.

Rota-rod: Motor functions were tested using the Rota-Rod test (Med Associates, St Albans, VT,) as previously described [3]. Briefly, animals were trained on a rotating drum and tested the following day with increasing velocity. Measures were taken of the duration each animal was able to maintain its balance walking on the rotating drum. The latency to fall was taken as a measure of motor function.

Open-field activity: The Activity Monitor system from Medical Associates (Med Associates, St Albans, VT,) was used to record locomotor activity as published previously [3]. Briefly, each subject was placed in the center of the open-field and activity was measured for 30 minutes and was recorded via a camera and stored for offline analysis.

Elevated plus maze test: The elevated plus maze (Med Associates, St Albans, VT,) consists of two open arms and two closed arms situated opposite each other and separated by a 6 cm square center platform. Each runway is 6 cm wide and 35 cm long. For each test, the animal was placed in the center square and allowed to move freely for five minutes. Open arm entries were defined as the mouse having all four paws onto the open arm. The number of entries and time spent in each arm was recorded.

Light/Dark emergence task: The Light/Dark test was performed as previously [3]. Briefly, the apparatus is a modified chamber (40 × 15.9 × 21.3 cm) separated into two compartments with a small opening (3.5 × 6 cm) between compartments. One compartment is completely dark, and the other is very bright. Mice were individually placed into the dark chamber of the box with the exit blocked for 5 seconds, after which the door lifted open and the mice were allowed to freely explore either compartment for 10 minutes. Time spent in the light chamber and light/dark compartment transitions were recorded.

## Results

### KChIP1 expression in the adult mouse brain

**Figure 1:**
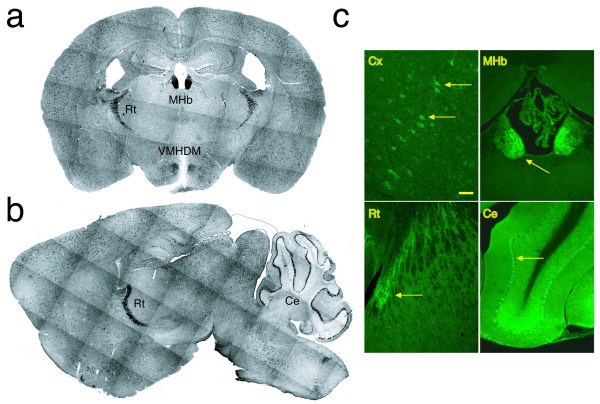
**KChIP1 expression in the adult mouse brain**. *a, b: In situ *hybridization. Adult mouse brain sections were hybridized *in situ *with an anti-sense RNA probe synthesized using KChIP1 cDNA as the template. Coronal (*a*) and sagittal (*b*) views are shown. Hybridization signals are seen in a subset of neurons distributed in the cerebral cortex, hippocampus, and thalamus. High expression of KChIP1 was detected in the medial habenular nucleus (*a*, MHb), the reticular thalamic nucleus (*a *and *b*, Rt), the dorsomedial part of the ventromedial hypothalamic nucleus (*a*, VMHDM), and the Purkinje cell layer of the cerebellum (*b*, Ce). The montage was produced from photographs taken with a deconvolution microscope using a 5× objective and Slidebook software (Intelligent Imaging Innovations, Inc.). *c: *Immunohistochemical detection of KChIP1 in the brain. Adult mouse brain sections were immunostained with a polyclonal anti-KChIP1 antibody. Expression of KChIP1 was analyzed under confocal microscopy. The distribution of KChIP1 protein was similar to that of KChIP1 mRNA detected by *in situ *hybridization. KChIP1 was detected in a subpopulation of neurons in the cerebral cortex (Cx, *yellow arrow*); high levels of KChIP1 were observed in the medial habenular nucleus (MHb, *yellow arrow*), reticular thalamic nucleus (Rt, *yellow arrow*), and Purkinje cell layer of the cerebellum (Ce, *yellow arrow*). Bar = 50 μm in Cx and 100 μm in MHb, Rt, and Ce.

The expression of KChIP1 in the adult mouse brain was initially investigated by *in situ *hybridization. Two probes were synthesized using KChIP1 cDNA as templates. One included the 3' UTR and partial coding sequences, while the other consisted of the 5'UTR and partial coding sequences. Similar results were obtained using both probes. Consistent with previous reports, KChIP1 expression was detected in multiple regions of the adult brain, including cortex, hippocampus, thalamus, hypothalamus, and cerebellum (Fig 1a and 1b) [2,9]. Individual KChIP1-positive neurons were found in all layers of the cerebral cortex. KChIP1-expressing neurons were scattered in the hippocampal CA1-3 region as well as in the dentate gyrus. In the cerebellum, KChIP1 was detected in the Purkinje cell layer only. A faint KChIP1 hybridization signal was observed in the molecular and granular layers of the cerebellum. In the hypothalamus, KChIP1-positive cells were concentrated in dorsomedial regions of the ventromedial hypothalamic nucleus. Only a few labeled cells were scattered in the ventrolateral and posterior hypothalamic areas. Interestingly, KChIP1 is expressed very highly in the medial habenular nucleus and the reticular thalamic nucleus. Hybridization signals were also seen in a small population of neurons in the oriens, pyramidale, and radiatum of the striatum. No significant hybridization signal was detected in the KChIP1 deficient mouse brain. Hybridization with sense probes generated only a faint background signal, confirming the specificity of the antisense probes (not shown).

To examine the localization of KChIP1 protein in the brain, we raised a polyclonal antibody against KChIP1 in rabbit. In agreement with the *in situ *hybridization results, immunohistochemical detection revealed KChIP1 expression in a subpopulation of neurons in the cerebral cortex, hippocampus (not shown), and in the entire cerebellar Purkinje cell layer, reticular thalamic nucleus, and medial habenular nucleus (Fig. 1c). Similar results were observed in rat and mouse. Thus, KChIP1 is expressed in a subpopulation of neurons located in multiple regions of the adult mammalian brain.

### Synaptic localization of KChIP1 in a subset of parvalbumin-positive GABAergic neurons

**Figure 2:**
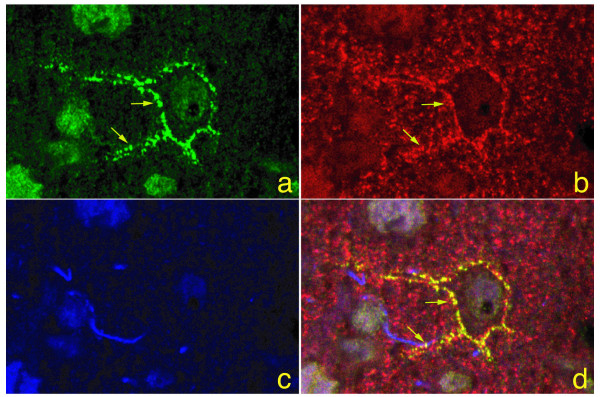
**Synaptic localization of KChIP1 in a subset of parvalbulmin-positive GABAergic *neurons presynaptically in vivo***. *a: *Adult rat cortex was immunostained with a polyclonal antibody against KChIP1 (*green*), and *b: *a monoclonal antibody against synaptophysin (*red*), and *c: *a monoclonal antibody against GFAP (*blue*). Sections were examined under confocal microscopy. *d: *An overlay image shows colocalization (*yellow*) of KChIP1 and synaptophysin at the plasma membrane of neuronal cell bodies and processes. No GFAP-labeled cells were KChIP1 positive. Arrows indicate colocalization of KChIP1 and synaptophysin. Bar = 40 μm.

We have previously shown KChIP1 is predominantly expressed by a subpopulation of parvalbumin-positive GABAergic neurons[33]. However, an exception to this rule occurred in the medial habenular nucleus, where neurons were decorated by the anti-KChIP1 antibody but not the anti-parvalbumin antibody (Fig. 1b and 1c). The medial habenular nucleus consists of mostly cholinergic neurons [34]. We further examined the subcellular localization of KChIP1 using both monoclonal (not shown) and polyclonal antibodies. In primary rat cerebrocortical cultures, KChIP1 was visualized in a subset of neurons (<10%) at synaptic bouton-like structures that clustered at the plasma membrane of both cell bodies and processes. Staining was abolished by pre-absorption of the antibodies with a KChIP1-GST fusion protein but not with GST protein alone. KChIP1-positive structures were co-labeled with antibodies against synaptophysin and synaptic vesicle protein 2 (SV2) (not shown), consistent with a presynaptic localization of KChIP1. Moreover, immunohistochemical analysis of adult rat brain revealed colocalization of KChIP1 and synaptophysin in a punctate pattern on the plasma membrane (Fig 2). GFAP-positive cells did not label for KChIP1 (Fig 2c). These results indicate a predominantly neuronal expression of KChIP1 in the adult brain and further verify the presynaptic localization of KChIP1 *in vivo*.

### Overexpression of KChIP1 facilitates GABA-mediated synaptic transmission in cultured hippocampal neurons

**Figure 3:**
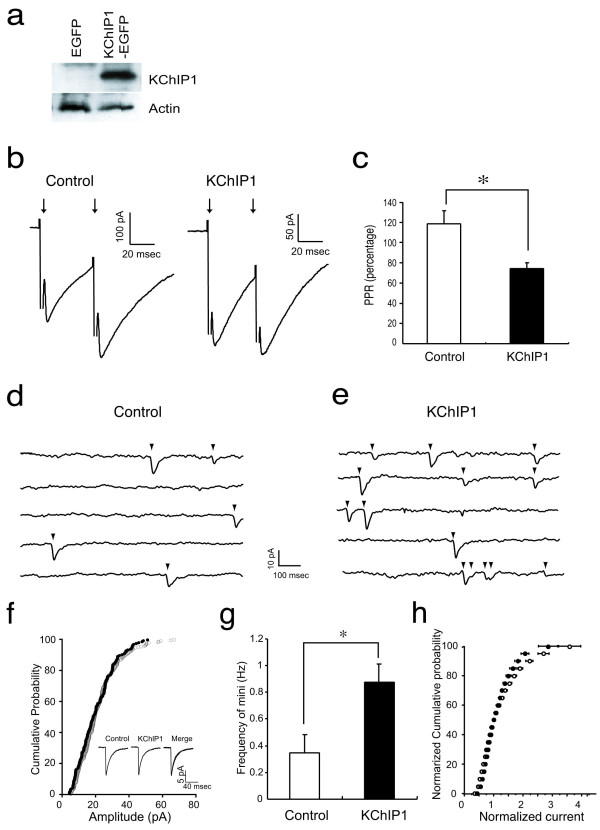
**Over-expression of KChIP1 facilitates GABA-mediated synaptic transmission in cultured hippocampal neurons**. *a*: Expression of KChIP1-EGFP was detected by immunoblot analysis in neurons infected with KChIP1-EGFP (KChIP1) but not in neurons infected with EGFP alone. Detection of actin in the lysates with an anti-actin antibody is shown as a loading control. *b*: Representative recordings from EGFP+ (Control) and KChIP1-EGFP neurons (KChIP1) are shown. *c*: The paired pulse ratio (PPR) recorded in control cells was significantly larger than that seen in KChIP1-expressing cells (*P < 0.01). *d, E*: mIPSCs from autaptic inhibitory neurons. Representative mIPSC recordings are shown from EGFP+ (*d*) and KChIP1-EGFP+ neurons (*e*). *f*: Cumulative probability of amplitude and averaged mIPSCs (*inset*) from the same two cells as on *f *and *e. g*: The frequency of mIPSCs in cells expressing KChIP1 (0.88 ± 0.14 Hz) was more than twice that observed in control cells expressing EGFP alone (0.37 ± 0.13; *P < 0.05). *h*: There was no difference in the amplitude distribution of mIPSCs in these neurons (KChIP-EGFP: filled dots; EGFP alone: empty dots).

We investigated the role of KChIP1 in modulating inhibitory synaptic transmission in cultured hippocampal neurons infected with recombinant Semliki Forest viral (SFV) particles encoding either a KChIP1-EGFP fusion protein or EGFP protein alone. Expression of KChIP1-EGFP was confirmed by immunoblotting with an anti-KChIP1 antibody (Fig. 3a). To assess its effect on physiological activity, we overexpressed KChIP1 in this neuronal cell type because only a subset of hippocampal neurons expressed endogenous KChIP1 and at relatively low levels. Using patch electrodes, we recorded autaptic GABA-mediated IPSCs from isolated EGFP-labeled neurons. IPSCs in these cultures were mediated exclusively by GABA_A _receptors that were blocked by the antagonists bicuculline or picrotoxinin [35]. Expression of KChIP1-EGFP had no significant effect on the decay time constants of autaptic GABA IPSCs (48.0 ± 6.7 ms, *n *= 7 for EGFP; 35.7 ± 3.0 ms, *n *= 8 for KChIP1-EGFP; *P *> 0.1 by Student's *t*-test). The peak amplitude of the IPSCs was 373 ± 108 pA (*n *= 8) in KChIP1-EGFP-positive neurons, while it was 233 ± 67.2 pA (*n *= 7) in EGFP-labeled neurons. Unlike the amplitude of GABA-mediated IPSCs, paired pulse facilitation is independent of postsynaptic receptor density or the number of synapses [36]. Here, we examined the effect of KChIP1 expression on this purely presynaptic phenomenon. Paired stimuli at intervals of 40 ms resulted in a 119 ± 12.8% increase in the amplitude of the second autaptic GABA IPSC in neurons expressing EGFP alone (*n *= 7). In contrast, for KChIP1-EGFP neurons, the paired pulse ratio was substantially reduced (74 ± 5.7%, *n *= 8; *P *< 0.01). This finding was consistent with the notion that KChIP1-EGFP expression increased the presynaptic release probability of GABA (Fig.3b and 3c). In agreement with the paired pulse facilitation results, the frequency of spontaneous autaptic miniature IPSCs (mIPSCs) in neurons expressing KChIP1-EGFP (0.88 ± 0.14 Hz, *n *= 12) was significantly higher than that seen in neurons expressing EGFP alone (0.37 ± 0.13 Hz, *n *= 10; *P *< 0.05) (Fig. 3d,e and 3g). However, there was no difference in the mean amplitude or amplitude distribution of mIPSCs in neurons expressing KChIP1-EGFP and control EGFP-positive neurons (Fig 3f and 3h). These results suggest that KChIP1 potentiates GABA-mediated inhibitory synaptic transmission via an increase in presynaptic GABA release.

### KChIP1 inhibits potassium currents in cultured hippocampal neurons

**Figure 4:**
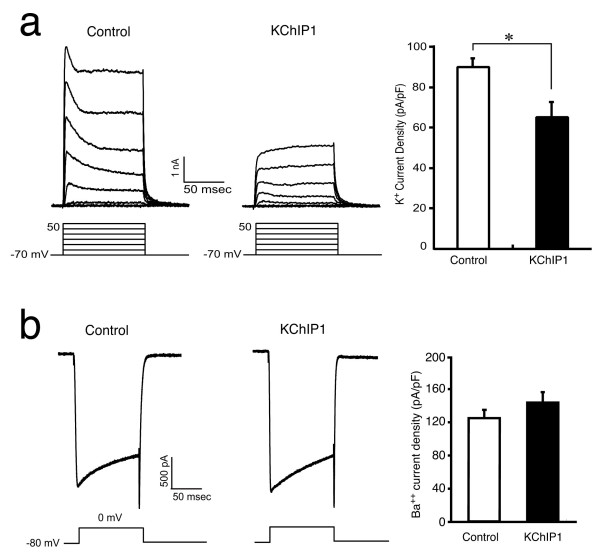
**KChIP1 decreases potassium but not calcium current densities**. *a*: Inhibition of potassium currents by KChIP1. Representative recordings from control neurons expressing EGFP alone or KChIP1-EGFP. Potassium current density in KChIP1+ neurons was significantly smaller than that seen in control (*P < 0.01, *n *= 18). Whole-cell potassium currents were evoked by a voltage step to -15 mV from a holding potential of -75 mV. Steady-state amplitudes were normalized to cell capacitance. *b *Expression of KChIP1 does not affect Ca^2+ ^channels. Representative recordings from control (EGFP+) and KChIP1+ (KChIP1-EGFP) neurons. Ba^2+ ^current density in KChIP+ neurons (143.17 ± 11.1 pA/pF, *n *= 11) was similar to that observed in controls (124.3 ± 8.9 pA/pF, *n *=12; *P > 0.1). Whole cell Ba^2+ ^currents were evoked by a voltage step to 0 mV from a holding potential of -80 mV.

To study the possible mechanism of KChIP1-induced presynaptic potentiation, we monitored whole-cell potassium and calcium currents in cultured hippocampal neurons infected with either KChIP1-EGFP or EGFP constructs (Fig. 4a, b). To minimize the effect of cell size, currents were normalized to neuronal membrane capacitance. Potassium current density in neurons expressing KChIP1-EGFP was significantly smaller (65 ± 7.6 pA/pF, *n *= 9) than in EGFP-expressing neurons (93.3 ± 2.9 pA/pF, *n *= 9; *P *< 0.01). In contrast, KChIP1 exhibited no significant effect on calcium current density. These results suggest that KChIP1 modulates neuronal potassium channels but not calcium channels under these conditions. Of note, the K^+ ^current recorded in this study is largely non-inactivating in nature, indicating that a non-A-type K^+ ^channel is predominantly involved.

**Figure 5:**
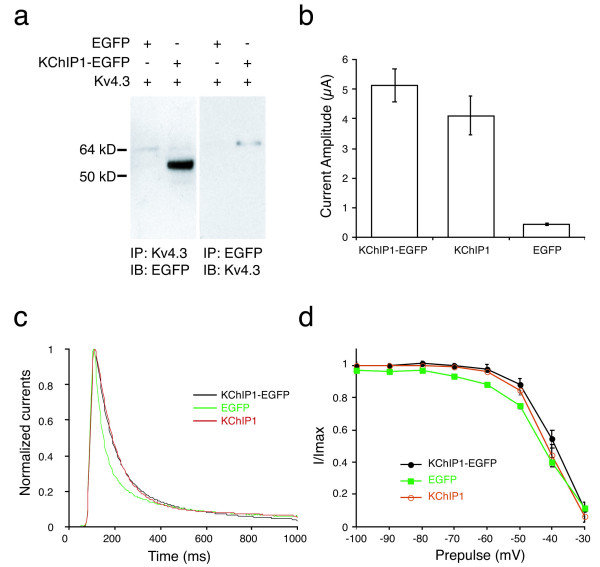
**KChIP1 inhibits potassium currents in cultured hippocampal neurons**. *a*: Co-immunoprecipitation of Kv4.3 and KChIP1-EGFP. HEK293 cell lysates co-expressing Flag-tagged Kv4.3 and KChIP1-EGFP were immunoprecipitated with an anti-Flag antibody (*left panel*; IP: Kv4.3) or an anti-EGFP antibody (*right panel*; IP: EGFP) followed by immunoblotting with anti-EGFP (*left panel*; IB: EGFP) or anti-Flag (*right panel*; IB: Kv4.3), respectively. *b: *Potentiation of Kv4.3 current density by Kv4.3-EGFP in Xenopus oocytes. Kv4.3 was co-expressed with either KChIP1-EGFP fusion protein, KChIP1, or EGFP in Xenopus oocytes. KChIP1-EGFP and KChIP1 but not EGFP significantly increased Kv4.3 current density (*P < 0.001). *c, d*: KChIP1-EGFP modulates inactivation of Kv4.3 in Xenopus oocytes. Kv4.3 was co-expressed with either KChIP1-EGFP fusion protein (*black line*), KChIP1 (*red line*), or EGFP (*green line*) in Xenopus oocytes. Expression of KChIP1-EGFP or KChIP1 enhanced inactivation of Kv4.3 from both the open state (*c*) and the closed state (*d*) compared with EGFP alone.

KChIP1 was previously demonstrated to potentiate Kv4 (A-type, fast inactivating) potassium channels in Xenopus oocytes [7]. In contrast, our results indicate the opposite effect of KChIP1 on non-inactivating potassium channels in cultured rat neurons. In order to ensure that our findings were not due to the EGFP moiety that was used to tag KChIP1 in our expression vector rather than KChIP1 itself, we compared the properties of KChIP1 and KChIP1-EGFP proteins. KChIP1-EGFP fusion protein co-immunoprecipitated with Kv4.3 in HEK293 cells co-transfected with cDNAs encoding KChIP1-EGFP and Kv4.3 (Fig 5a). We found in Xenopus oocytes that Kv4.3 current density was potentiated by expression of either KChIP1-EGFP fusion protein (to 5.1 ± 1.7 μA/pF, *n *= 10) or KChIP1 alone (to 4.1 ± 2.0 μA/pF, *n *= 9). Potentiation could be attributed to a slowing of channel inactivation, as previously demonstrated for KChIP1 (Fig 5c and 5d). No significant difference in the potency of modulation of Kv4.3 channels was found between KChIP1-EGFP fusion protein and KChIP1. These results show that KChIP1-EGFP fusion protein and KChIP1 function similarly. These results are further supported by the recent findings that GFP-tagged KChIP1 functionally stimulates Kv4.2 trafficking in transfected cells [27,37]. Hence, our results demonstrating a difference between the effects of KChIP1 on potassium channel modulation in Xenopus oocytes and in neurons cannot be attributed to differing effects of KChIP1-EGFP and KChIP1.

### Disruption of mouse KChIP1 gene potentiates potassium currents in cultured Purkinje neurons

**Figure 6:**
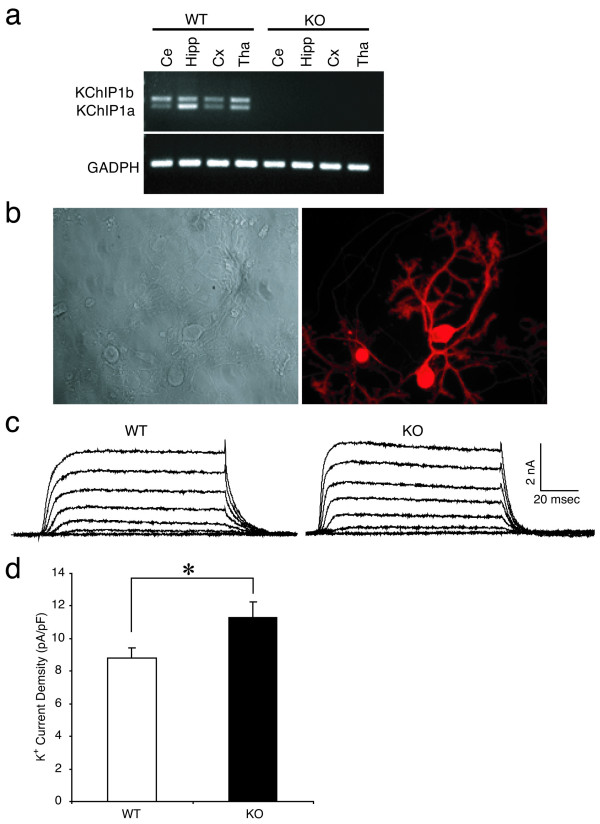
**Disruption of mouse KChIP1 gene potentiates potassium currents in cultured Purkinje neurons**. *a: *Lack of KChIP1 mRNA in KChIP1^-/- ^mouse brain tissue. KChIP1a and KChIP1b mRNAs in wild-type (WT) and KChIP1^-/- ^(KO) mouse brain were analyzed by RT-PCR. The brain tissues examined include cerebellum (Ce), hippocampus (Hipp), cortex (Cx) and thalamus (Tha). Expression of GAPDH was monitored as a positive control. *b*: Primary cultures of Purkinje cells from KChIP1^-/- ^mouse cerebella. A phase contrast image (*left panel*) and an immunofluorescence image of Purkinje cells stained by with anti-calbindin antibody (*right panel*) are illustrated. *c, d*: Potentiation of potassium currents in KChIP1^-/- ^Purkinje cells. Representative recordings from wild-type (WT) and KChIP1^-/- ^(KO) Purkinje cells (*c*). Potassium current density in WT was significantly smaller than that seen in KO neurons (*d*, *P < 0.05). Whole-cell potassium currents were evoked by a voltage step to -15 mV from a holding potential of -75 mV. Steady-state amplitudes were normalized to cell capacitance.

To further investigate KChIP1 function *in vivo*, we generated mice with a genetic disruption of the KChIP1 gene. Expression of KChIP1a and KChIP1b, two reported KChIP1 splice variants [7,38], are ablated in the brains of these knockout (KO) mice (Fig. 4a). Since Purkinje neurons represent neurons that express high levels of KChIP1 and are morphologically identifiable in culture, we compared potassium currents in cultured Purkinje neurons generated from KChIP1 wild-type (WT) mice and KChIP1 KO mice. Cultured mouse Purkinje cells were morphologically identified under microscopy and immuno-verified with anti-calbindin antibody staining after electrophysiological recording (Fig. 6b). Potassium currents were normalized to neuronal membrane capacitance. We found that potassium current density in KChIP1 KO Purkinje cells was significantly larger (11.3 **± **0.95 pA/pF, *n *= 13) than in WT Purkinje neurons (8.8 **± **0.60 pA/pF, *n *= 13; *P *< 0.05) (Fig. 6c and 6d). Thus, ablation of KChIP1a and KChIP1b resulted in increased potassium current density in Purkinje cell neurons. These findings are consistent with those in neurons overexpressing KChIP1-EGFP and provide further evidence that KChIP1 inhibits neuronal potassium channels *in vivo*.

### KChIP1 KO mice display enhanced anxiety-like behavior

**Figure 7:**
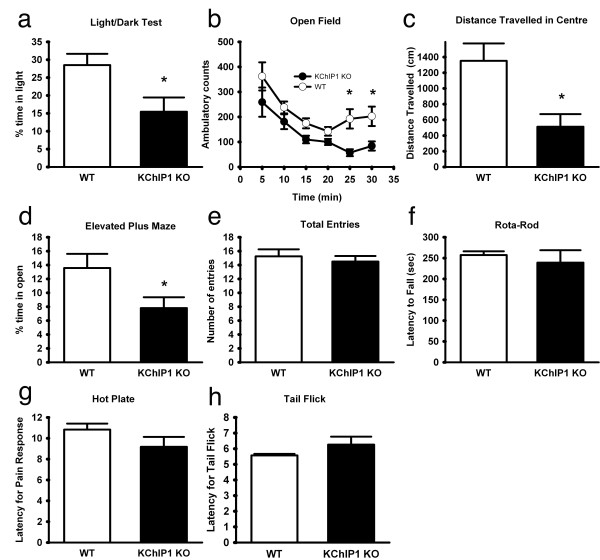
**KChIP1 KO mice display enhanced anxiety-like behavior**. *a*: In the light/dark test, KChIP1 KO mice (n = 6) spent significantly less time in the light compartment compared to WT controls (n = 5) (*P < 0.05.). *b*: In the open field test, KChIP1 KO mice had significantly less ambulatory counts compared to WT controls (n = 7). (* *P *< 0.02). *c: *KChIP1 KO mice (n = 9) travelled significantly less within the centre area compared to WT controls (n = 7). *d*: In the elevated plus maze test, KChIP1 KO mice (n = 9) spent significantly less time in the open arms to WT mice (n = 7), however *e*: KChIP1 KO mice (n = 9) made similar amount of entries into the arms compared to WT controls (n = 7) (* *P *< 0.05). *f*: In a rota-rod test for motor performance, KChIP1 KO mice (n = 6) exhibited similar falling latencies as WT control mice (n = 6). *g*: On a thermal hot plate test, KChIP1 KO mice (n = 6) displayed similar latencies for nociceptive behavior display as WT control mice (n = 6). *h*: In a tail flick assay, KChIP1 KO mice (n = 6) displayed similar latencies for tail flicks as WT control mice (n = 6).

Given our observations that KChIP1 potentiates presynaptic GABA release and that benzodiazepines, commonly used for the relief of anxiety, are thought to act by enhancing the action of the inhibitory transmitter GABA, we investigated the possible role of KChIP1 in anxiety-like behavior and compared KChIP1 KO and WT mice in a battery of anxiety related tasks. In the light/dark emergence task, where a box is separated into light and dark compartments, KChIP1 KO mice spent a significantly smaller percentage of the time in the light compartment (17.72 ± 3.98 sec, *n *= 6) compared to WT mice (28.51 ± 3.16 sec, *n *= 5; *P *< 0.05) (Fig 7a) and also made significantly fewer crossings between compartments (14.33 ± 2.75, *n *= 6) compared with WT mice (38.8 ± 5.08 sec, *n *= 5; *P *< 0.005) (not shown). In the open field paradigm, KChIP1 KO mice displayed significantly less activity after 25 minutes (57 ± 10.92 sec, *n *= 9) and 30 minutes (83.33 ± 15.02 sec, *n *= 9) compared to WT mice (192.67 ± 27.92 sec, *n *= 9; *P *< 0.005) and (202.43 ± 38.92 sec, *n *= 9 *P *< 0.02) respectively (Fig. 7b). Furthermore, KChIP1 KO mice travelled significantly less in the centre area (512.14 ± 160.64 sec, *n *= 9) compared to control mice (1351.75 ± 221.55 sec; *n *= 7; *P *< 0.05) (Fig. 7c). In the elevated plus maze task, KChIP1 KO mice spent a significantly smaller percentage of the time in the open arms (7.81 ± 1.56 sec, *n *= 9) compared to WT mice (13.57 ± 2.04 *n *= 7; *P *< 0.05) (Fig. 7d). Importantly, total number of entries did not differ between KChIP1 KO and WT mice (Fig 7e), confirming that the results are not due to alterations in motor ability.

### KChIP1 KO mice do not display sensory or motor deficits

KChIP1 KO mice (239.11 ± 72.69 sec, *n *= 6) and WT mice (257.22 ± 22.06 sec, *n *= 6) displayed similar fall latencies in the rota-rod test for motor performance (Fig 7f). Additionally, KChIP1 KO showed similar latencies for nociceptive response in the hot plate test (9.18 ± 2.33 sec, *n *= 6) compared to WT mice (10.83 ± 1.42 sec, *n *= 6) (Fig 7g). Similarly, KChIP1 KO and control mice showed similar latencies for tail flick responses (6.26 sec ± 1.24 sec, *n *= 6) and (5.57 ± 0.24 sec, *n *= 6) respectively (Fig. 7h).

## Discussion

In this study, we employed two independent experimental approaches, *in situ *hybridization and immunohistochemical staining, to detect KChIP1 in the adult mammalian brain. We found that KChIP1 is expressed in a subpopulation of neurons widely distributed in the cortex, thalamus, hypothalamus, hippocampus, and amygdala. Additionally, high expression of KChIP1 in the cerebellar Purkinje cell layer and the thalamic reticular nucleus suggested an association with inhibitory neurons. In support of this notion, neurons expressing KChIP1 were also found to be parvalbumin-positive. Our results are consistent with a recent finding of KChIP1 expression in interneurons in the rat brain [2]. Parvalbumin-containing neurons constitute the largest subset of inhibitory GABAergic interneurons in the brain [39]. These neurons are often fast spiking and morphologically heterogeneous [40,41]. The subset of parvalbumin-positive, GABAergic cells expressing KChIP1 may represent a functionally distinct pool of inhibitory neurons. To test this idea, we investigated the physiological effect of KChIP1 in central neurons. Most importantly, we found three lines of evidence that suggest a role for KChIP1 in enhancing inhibitory synaptic transmission via a presynaptic mechanism. First, KChIP1 localized to presynaptic structures in parvalbumin-expressing neurons. Second, expression of KChIP1-EGFP in cultured hippocampal neurons reduced the degree of paired pulse facilitation observed in GABA-mediated IPSCs. Finally, expression of KChIP1-EGFP increased the frequency but not the amplitude of mIPSCs.

These findings can be compared to those on the KChIP1 homologues NCS-1 and frequenin. Frequenin, a *Drosophila *homologue of KChIP1, is enriched at synapses. Transgenic flies overexpressing frequenin exhibit frequency-dependent facilitation of neurotransmitter release at the neuromuscular junction [10]. In *C. elegans*, NCS-1 regulates associative learning and memory [11]. Recent studies suggest that mammalian NCS-1 contributes to activity-dependent synaptic facilitation at presynaptic nerve terminals via an increase in calcium current [12]. Thus, other neuronal calcium sensor proteins appear to be involved in modulating synaptic efficacy and plasticity[42]. However, despite our evidence for a presynaptic effect of KChIP1, our results do not totally exclude the possibility of an additional postsynaptic function of KChIP1, for example, by modulating Kv4 channels, which may be predominately postsynaptic in localization. In fact, KChIP1 and Kv4.3 are colocalized along the somato-dendritic membranes of interneurons [2].

Here we explored the underlying mechanism of facilitation of inhibitory synaptic transmission by KChIP1. Intriguingly, previous studies had suggested that KChIP1 increases potassium channel activity in non-neuronal cells, including Xenopus oocytes and CHO cells [8,18,23]. If this were the mechanism of action in neurons, however, KChIP1 would inhibit rather than facilitate synaptic transmission, as we found. In contrast to other NCS proteins, our studies suggest that KChIP1 facilitates inhibitory synaptic transmission in neurons by inhibiting potassium channel activity rather than by enhancing calcium channel currents. Along these lines, we report here for the first time that overexpression of KChIP1 inhibits whole-cell K^+ ^currents in cultured hippocampal neurons. Consistent with this notion, genetic ablation of KChIP1 potentiates potassium currents in Purkinje cells. In addition, unlike NCS-1, overexpression of KChIP1 had no direct effect on whole-cell calcium currents. It remains unclear, however, why KChIP1 exhibits opposite effects on potassium currents in neurons and in non-neuronal cells. The most likely explanation is that different types of potassium channels are involved; prior studies found that KChIP1 facilitated Kv4 A-type (fast inactivating) K^+ ^currents, whereas we observed that KChIP1 inhibited non-inactivating K^+ ^currents. Along these lines, it was recently reported that KChIP1 and KChIP2 inhibit trafficking of non-inactivating Kv1.5 K^+ ^channels, thus decreasing their insertion into the plasma membrane [43]. This finding offers a possible mechanism for our physiological observations.

Recent studies suggest that parvalbumin-expressing neurons constitute a subpopulation of GABAergic neurons that are vulnerable in several neurodegenerative and neuropsychiatric disorders. In the brains of Huntington's disease patients, for example, and mice expressing mutant huntingtin protein, a large percentage of parvalbumin-positive neurons selectively degenerate [44,45]. Severe loss of parvalbumin-positive neurons is also observed in human transmissible spongiform encephalopathies (TSEs), prion diseases[46,47]. Furthermore, a profound and selective loss of parvalbumin-positive neurons is found in the hippocampus of patients with schizophrenia, bipolar disorder and epilepsy[48-52]. In fact, it has been suggested that impaired synaptic transmission may contribute, at least in part, to the loss of these neuronal populations [53,54]. Furthermore, benzodiazepines, commonly used for the relief of anxiety, have long been understood to act by enhancing GABAergic activity, although the mechanisms involved in this effect are not yet fully understood. Here we report that KChIP1 KO mice exhibited enhanced anxiety-like behavior across a battery of anxiety related tasks. They displayed avoidance of high, exposed, well light open areas, and displayed less exploratory activity than WT mice. These observations are consistent with the anxiolytic effects of GABA receptor stimulation, and suggest that KChIP1-dependent potentiation of presynaptic GABA release is a mediator of anxiety. Importantly, KChIP1 KO mice did not exhibit deficiencies in nociception or motor functions, indicating that KChIP1 protein may be selectively involved in the perception of anxiety-related behavior. Given its expression in parvalbumin-expressing neurons and its role in enhancing inhibitory synaptic transmission, it will be important to further investigate the potential role of KChIP1 in mental health and neurodegenerative diseases.

## Conflict of interests

The authors declare that they have no competing interests.

## Authors' contributions

KX, HX, YS, DLW, TD, HT, and GD performed the experiments included in the manuscript. MHE, SAL, GT, DZ, MZ, and ZZ conceived of the experiments. MZ and ZZ wrote the manuscript. All of the authors have read the manuscript.
